# Artificial Intelligence-Based Automated Treatment Planning of Postmastectomy Volumetric Modulated Arc Radiotherapy

**DOI:** 10.3389/fonc.2022.871871

**Published:** 2022-04-25

**Authors:** Shengpeng Jiang, Yi Xue, Ming Li, Chengwen Yang, Daguang Zhang, Qingxin Wang, Jing Wang, Jie Chen, Jinqiang You, Zhiyong Yuan, Xiaochun Wang, Xiaodong Zhang, Wei Wang

**Affiliations:** ^1^ Department of Radiation Ocology, Tianjin Medical University Cancer Institute and Hospital, National Clinical Research Center for Cancer, Key Laboratory of Cancer Prevention and Therapy, Tianjin’s Clinical Research Center for Cancer, Key Laboratory of Breast Cancer Prevention and Therapy, Tianjin Medical University, Ministry of Education, Tianjin, China; ^2^ Department of Radiation Physics, University of Texas MD Anderson Cancer Center, Houston, TX, United States

**Keywords:** artificial intelligence, automated treatment planning, postmastectomy radiotherapy (PMRT), volumetric modulated arc therapy (VMAT), MD Anderson Cancer Center AutoPlan (MDAP)

## Abstract

As a useful tool, artificial intelligence has surpassed human beings in many fields. Artificial intelligence-based automated radiotherapy planning strategies have been proposed in lots of cancer sites and are the future of treatment planning. Postmastectomy radiotherapy (PMRT) decreases local recurrence probability and improves overall survival, and volumetric modulated arc therapy (VMAT) has gradually become the mainstream technique of radiotherapy. However, there are few customized effective automated treatment planning schemes for postmastectomy VMAT so far. This study investigated an artificial intelligence based automated planning using the MD Anderson Cancer Center AutoPlan (MDAP) system and Pinnacle treatment planning system (TPS), to effectively generate high-quality postmastectomy VMAT plans. In this study, 20 patients treated with PMRT were retrospectively investigated, including 10 left- and 10 right-sided postmastectomy patients. Chest wall and the supraclavicular, subclavicular, and internal mammary regions were delineated as target volume by radiation oncologists, and 50 Gy in 25 fractions was prescribed. Organs at risk including heart, spinal cord, left lung, right lung, and lungs were also contoured. All patients were planned with VMAT using 2 arcs. An optimization objective template was summarized based on the dose of clinical plans and requirements from oncologists. Several treatment planning parameters were investigated using an artificial intelligence algorithm, including collimation angle, jaw collimator mode, gantry spacing resolution (GSR), and number of start optimization times. The treatment planning parameters with the best performance or that were most preferred were applied to the automated treatment planning method. Dosimetric indexes of automated treatment plans (autoplans) and manual clinical plans were compared by the paired t-test. The jaw tracking mode, 2-degree GSR, and 3 rounds of optimization were selected in all the PMRT autoplans. Additionally, the 350- and 10-degree collimation angles were selected in the left- and right-sided PMRT autoplans, respectively. The uniformity index and conformity index of the planning target volume, mean heart dose, spinal cord D_0.03cc_, mean lung dose, and V_5Gy_ and V_20Gy_ of the lung of autoplans were significantly better compared with the manual clinical plans. An artificial intelligence-based automated treatment planning method for postmastectomy VMAT has been developed to ensure plan quality and improve clinical efficiency.

## Introduction

Radiotherapy makes use of radiation to kill tumors, and it is one of the main methods of standard treatment of tumors. Treatment planning is a key step in the process of radiotherapy for patients, and it is to create a treatment plan file for radiotherapy equipment to deliver radiation for patients by optimization and calculation. Because the structure and geometric relationship of each patient’s target volume, organs at risk and normal tissues, the prescribed dose of the target volume, and the dose constraints of the organs at risk are probably different, it is necessary to seek a good balance for each treatment plan in terms of the dose of the target volume, organs at risk and normal tissue, plan complexity, dose calculation accuracy, treatment planning time, etc., which belongs to the category of multi-objective optimization problems, and manual decision-making and operation of the treatment planning system (TPS) by radiotherapy dosimetrists is usually required. Automated treatment planning generally refers to the application of an algorithm program, instead of manual decision-making and operation, to control the optimization and calculation of the TPS, and to realize the automated generation of treatment plans. In recent years, artificial intelligence technology has developed rapidly and has reached or surpassed the level of human processing tasks in many fields (1, 2). Automated treatment planning methods for multiple cancer sites have been proposed and implemented, and artificial intelligence-based automated planning has become a trend in the treatment planning (3–5).

Breast cancer is the most common malignant tumor in women (6), and breast cancer patients with T4 stage, axillary metastatic lymph nodes ≥4, or primary tumor diameter >5 cm are significantly benefited by postmastectomy radiotherapy (PMRT) (7, 8). Due to the large target volume, the chest wall target volume close to the heart, ipsilateral lung, and skin, strict requirements for dose uniformity and high-dose conformity of the target volume, and low-dose volume to organs at risk, the treatment planning is difficult. In some cases, the internal mammary region needs to be included (8), which increases the difficulty of treatment planning. It is time-consuming and laborious to complete the treatment plan manually, and the plan quality is not easy to guarantee, so an automated treatment planning solution is needed.

Various irradiation techniques have been reported for PMRT, including fixed beam intensity-modulated radiotherapy (IMRT), volumetric modulated arc therapy (VMAT), TomoDirect, TomoHelical, and mixed photon and electron beam irradiation (9–13). Kisling et al. (14) implemented an automated treatment planning method similar to manual forward IMRT planning for left-sided PMRT. VMAT has gradually become the mainstream technique of radiotherapy due to its good capability of radiation modulation, short time of dose delivery, convenient operation of setup, and easy availability of radiotherapy equipment (15, 16). Cilla et al. (17) conducted a preliminary feasibility study of automated treatment planning of left-sided postmastectomy VMAT.

VMAT generally refers to an irradiation technique in which in the process of X-ray delivery of the accelerator, the rotation speed of the gantry around the isocenter is variable, the angles of the treatment couch and the collimation are fixed, the movement speed of the multi-leaf collimator (MLC) is variable, and the delivered dose rate is variable. The treatment planning of VMAT usually needs to be optimized and calculated by the radiotherapy dosimetrist using TPS. The rotation speed of the gantry, the movement of the MLC, and the dose rate of the radiation delivery could be optimized by the optimizer of TPS according to the optimization objectives set manually. However, some other beam parameters, such as the number of beams, range of gantry angle, collimation angle, jaw collimator mode and size limit, and gantry spacing resolution (GSR), as well as some optimization process-controlling parameters, such as the maximum iterations for each start optimization times, stopping tolerance, and number of start of optimization, need to be set manually, and the optimization objectives and the above various treatment planning parameters may need to be manually adjusted during the optimization process (18, 19).

The current research hotspot of IMRT-automated planning is the setting and adjusting of optimization objectives for different cancer sites, which mainly include the following. 1) The dose information of a new plan is predicted based on building a model of the plan library and fitting coefficients of the features or deep learning methods, which is converted into optimization objectives, and no adjusting is generally required during the optimization process (4, 20). 2) The optimization objectives are set based on the optimization objective template or dose information of a similar plan, which may need to be adjusted by the algorithm program during the optimization process (21, 22). However, there are few studies on treatment planning parameters such as other beam parameters and optimization process-controlling parameters of automated treatment planning, which are generally set manually according to experience in the algorithm program.

The MD Anderson Cancer Center AutoPlan (MDAP) system (21) provides treatment planners with an automated planning tool that enables one button click to generate treatment plans. The MDAP system provides the interface of the treatment planning language scripting. Treatment planners can write an MDAP program as the algorithm that controls the treatment planning process, which can dynamically generate and execute the scripts of TPS and control TPS to generate a treatment plan. In this study, the MDAP system and the Pinnacle (v9.8, Philips Radiation Oncology Systems, Fitchburg, WI) TPS were used to compare and select several beam parameters and optimization process-controlling parameters to propose and evaluate an artificial intelligence-based automated treatment planning method for postmastectomy VMAT.

## Materials and Methods

### Patient Selection and CT Simulation

20 cases were randomly selected from the patients who underwent postmastectomy VMAT in our institution from October 2018 to January 2019, including 10 cases of left-sided PMRT and 10 cases of right-sided PMRT. The patients were placed in supine position with free breathing and were immobilized using the thermoplastic mask (Klarity Medical & Equipment Co. Ltd., Guangzhou, China). The arm of the affected side was lifted upward and abducted, the arm of the unaffected side was placed at the side of the body, and the skin of the chest wall was covered with a 1-cm-thickness bolus. CT simulation images of the patients were acquired using a Philips Big Bore CT with the slice thickness of 5 mm and then transferred to the Pinnacle TPS.

### Delineation and Prescription Dose

Referring to the RTOG guidelines for delineating the target volume of PMRT, the target volume was delineated by an experienced senior radiation oncologist and reviewed by a superior radiation oncologist. The clinical target volume (CTV) is the chest wall and the supraclavicular, subclavicular, and internal mammary regions of the affected side. The planning target volume (PTV) is the CTV uniformly extended 5 mm in 3 dimensions, and it is extended into the bolus at the chest wall. Organs at risk were delineated including the heart, spinal cord, left lung, right lung, and lungs.

**Tables 1**, **2** list the volume data of the PTV and some organs at risk for left- and right-sided PMRT cases, respectively. The prescribed dose is 50 Gy in 25 fractions for the PTV, requiring V_50Gy_ > 90% and V_47.5Gy_ > 95% of the PTV.

**Table 1:** Structure volumes of left-sided PMRT cases.

Case no.	PTV	Left lung	Right lung	Heart	Overlap of PTV and left lung
1	967.4	928.8	1,334.3	497.7	59.3
2	1,086.4	992.2	1,343.8	609.6	78.9
3	1,283.1	771.3	1,224.8	642.4	59.6
4	1,504.6	805.2	984.5	565.5	37.5
5	1,322.3	1,114.3	1,180.7	621.9	70.1
6	1,035.5	916.5	1,212.4	664.8	55.2
7	1,104.8	1,050.5	1,359.7	503.3	72.8
8	1,280.9	1,132.8	1,423.5	657.7	79.6
9	1,402.0	708.1	955.5	651.1	50.7
10	1,139.0	866.0	1,095.6	620.4	60.2

Volumes were given in cc.

**Table 2:** Structure volumes of right-sided PMRT cases.

Case no.	PTV	Left lung	Right lung	Heart	Overlap of PTV and right lung
1	1,009.3	1,351.7	1,512.8	546.8	125.5
2	1,265.2	838.6	1,042.4	709.3	81.7
3	1,217.5	1,069.3	1,277.7	549.9	103.7
4	1,284.3	840.6	1,091.1	562.6	94.5
5	913.9	1,586.6	1,955.3	612.9	107.0
6	1,178.5	1,549.0	1,748.4	537.5	110.9
7	1,476.4	805.8	1,210.2	606.2	104.4
8	1,311.6	1,534.8	1,610.6	526.6	91.3
9	1,394.2	755.9	1,001.6	579.9	71.0
10	1,147.4	1,108.3	1,391.9	792.7	115.1

Volumes were given in cc.

### TPS, Radiotherapy Equipment, and Autoplan System

All VMAT plans for this study were optimized using the Pinnacle TPS, and the optimizer was SmartArc. The radiotherapy equipment adopts a TrueBeam (Varian Medical Systems, Palo Alto, CA) linear accelerator equipped with a Millennium 120 MLC, 6-MV photons, and a maximum delivery dose rate of 600 MU/min. The jaw collimator has two modes of jaw tracking and fixed jaw. Using the MDAP system, an algorithm that controls the treatment planning process can be written as an MDAP program, which can dynamically generate and execute the scripts of the Pinnacle TPS and control the TPS to generate a treatment plan, including generating auxiliary structures, setting optimization objectives, setting various treatment planning parameters, starting optimization, and adjusting optimization objectives.

### Auxiliary Structure Generation and Optimization Objective Setting

Since the range, shape, and prescribed dose of the target volume of PMRT are relatively uniform, and the positional relationship between the target volume and organs at risk is also relatively consistent, a template-based optimization objective setting method can be adopted for the automated treatment planning; that is, a universal optimization objective template is summarized based on the dose of clinical plans and requirements from oncologists that applies to all plans and does not need to be adjusted during the optimization process. **Table 3** shows the auxiliary structure name and generation method. **Table 4** shows the optimization objective template.

**Table 3:** Generation method of auxiliary structures.

Auxiliary structures	Generation method
PTV-3mm	Create contraction of PTV with a 3-mm margin
PTV 5mmring	Create ring of PTV with a 5-mm margin
PTV 1cmring	Create ring of PTV between the 5-mm and 1-cm margin
PTV 2cmring	Create ring of PTV between the 1-cm to 2-cm margin
PTV 3cmring	Create ring of PTV between the 2-cm to 3-cm margin
nt	Create subtraction of PTV and PTV rings from the body
Left lung avoid	Create subtraction of PTV and PTV 5mmring from left lung
Right lung avoid	Create subtraction of PTV and PTV 5mmring from right lung
Heart avoid	Create subtraction of PTV and PTV 5mmring from heart

**Table 4:** Optimization objective template of postmastectomy VMAT plans.

ROI	Type	Target cGy	% Volume	Weight	a
PTV	Max Dose	6,300		80	
PTV	Max Dose	6,300		80	
PTV	Max Dose	6,300		80	
PTV	Min DVH	6,000	98	100	
PTV	Min DVH	5,800	99	100	
PTV	Min Dose	5,500		100	
PTV-3mm	Min Dose	6,000		100	
PTV 5mmring	Max Dose	6,000		30	
PTV 1cmring	Max Dose	5,500		30	
PTV 2cmring	Max Dose	5,000		30	
PTV 3cmring	Max Dose	4,500		30	
nt	Max Dose	3,500		30	
Left lung	Max DVH	2,000	25	100	
Right lung	Max DVH	2,000	25	100	
Lungs	Max DVH	500	40	100	
PTV 1cmring	Max EUD	0		6e-09	1
PTV 2cmring	Max EUD	0		1e-08	1
PTV 3cmring	Max EUD	0		1.5e-08	1
nt	Max EUD	0		1e-07	1
Spinal cord	Max EUD	0		1e-08	10
Left lung avoid	Max EUD	0		1e-06	1
Right lung avoid	Max EUD	0		1e-06	1
Heart avoid	Max EUD	0		1e-06	1

### Setting of Some Treatment Planning Parameters

The isocenter is located in the box center of the PTV. Using dual arcs, the gantry angle range for the left-sided PMRT plan is 294 to 180 degrees, and the gantry angle range for the right-sided PMRT plan is 181 to 66 degrees. The second arcs are generated by the optimizer after fluence optimization with the same gantry angle ranges, the opposite gantry rotation orientations, and the same collimation angles as the first arcs. The maximum size limit for the movement of the jaw collimator in the x-direction is 10 cm left and 10 cm right. The dose calculation uses the collapsed cone convolution superposition algorithm, and the dose calculation resolution is 3 mm × 3 mm × 3 mm. The number of treatment fractions was set to 30 before optimization, changed to 25 after optimization, and normalized to satisfy V_50Gy_ > 90% and V_47.5Gy_ > 95%, and the beam MU was as small as possible. The maximum iterations for fluence optimization is 40, and the maximum iteration for each start of optimization is 100. The iteration stopping tolerance is that the difference between the objective function values of two adjacent iterations is less than 1e-6.

### Selection of the Collimation Angle

First, we make the selection of the collimation angle out of 5 degrees, 10 degrees, 350 degrees, and 355 degrees. The jaw collimator mode is set to jaw tracking, the GSR set to 2 degrees, and the optimization process is set to start optimization twice, that is, 2 rounds of optimization. Using the MDAP system, the above operations and settings are written as 4 MDAP programs, corresponding to 4 different collimation angles. For 20 cases, a total of 80 VMAT plans with collimation angles of 5 degrees, 10 degrees, 350 degrees, and 355 degrees were generated. The most preferred collimation angles were selected for the left- and right-sided PMRT plans, respectively, and statistical analysis was performed.

### Selection of Mode of Jaw Collimator

Based on the selected collimation angle, we choose the mode of the jaw collimator, including jaw tracking and fixed jaw. The collimation angle selected in the previous step is set, the GSR is set to 2 degrees, and the optimization process is set to start optimization twice. Using the MDAP system, the above operations and settings are written as 2 MDAP programs, corresponding to the jaw tracking and fixed jaw modes, respectively. For 20 cases, a total of 40 VMAT plans with the jaw collimator modes of jaw tracking and fixed jaw were generated. The most preferred modes of jaw collimator were selected for the left- and right-sided PMRT plans, respectively, and statistical analysis was performed.

### Selection of the GSR

Based on the selected collimation angle and the jaw collimator mode, we select the GSR, including 2 degrees and 4 degrees. The selected collimation angle and jaw collimator mode in the previous step are set, and the optimization process is set to start optimization twice. Using the MDAP system, the above operations and settings are written as 2 MDAP programs, corresponding to the GSR of 2 and 4 degrees, respectively. For 20 cases, a total of 40 VMAT plans with GSR of 2 and 4 degrees were generated. The most preferred GSRs were selected for the left- and right-sided PMRT plans respectively, and statistical analysis was performed.

### Selection of the Number of Start Optimization Times

Finally, we choose the number of start optimization times, including twice and 3 times, that is, 2 rounds and 3 rounds of optimization. The selected collimation angle, jaw collimator mode, and GSR in the previous step are set. Using the MDAP system, the above operations and settings are written as 2 MDAP programs, corresponding to 2 rounds and 3 rounds of optimization, respectively. For 20 cases, a total of 40 VMAT plans with 2 rounds and 3 rounds of optimization were generated. The most preferred starting optimization times were selected for the left- and right-sided PMRT plans, respectively, and statistical analysis was performed.

### Automated Treatment Planning

The selected collimation angle, jaw collimator mode, GSR, and start optimization times were set. Using the MDAP system, the above operations and settings are written as an MDAP program to realize the automated treatment planning function of postmastectomy VMAT. For 20 cases, a total of 20 VMAT-automated treatment plans (autoplans) were generated, and dosimetric comparison and statistical analysis were performed with the corresponding left- and right-sided PMRT manual clinical plans, respectively.

### Plan Evaluation

The dosimetric index includes the conformity index CI of the PTV, which is defined as


(1) $$CI=\frac{TV_{ref}}{TV}\times \frac{TV_{ref}}{V_{ref}}$$

where TV is the volume of the target volume, TV_ref_ is the volume of the target volume covered by the prescribed dose, and V_ref_ is all the volumes covered by the prescribed dose, and the larger the CI value, the better the conformity. The uniformity index HI of the PTV is defined as 


(2) $$HI=\frac{D_{2\%}-D_{98\%}}{D_{50\%}}$$

Among them, D_2%_, D_98%_, and D_50%_ represent the dose corresponding to 2%, 98%, and 50% of the PTV, respectively. The smaller the HI value, the better the uniformity. The mean heart dose (heart D_mean_), spinal cord D_0.03cc_, where D_0.03cc_ represents the dose corresponding to the 0.03-cc volume, mean dose, V_5Gy_ and V_20Gy_ of the ipsilateral lung (ipsilateral lung D_mean_, ipsilateral lung V_5Gy_, ipsilateral lung V20Gy), where V_5Gy_ and V_20Gy_ represent the volume corresponding to the 5- and 20-Gy doses, respectively, mean dose and V_5Gy_ of the contralateral lung, and mean dose of the lungs (lungs D_mean_) were evaluated.

### Statistical Analysis

Using SPSS software, the paired t-test method was used to compare and evaluate the plans of collimation angle selection, the plans of jaw collimator mode selection, the plans of GSR selection, the plans of start optimization times selection, and the automated plans and manual clinical plans respectively. p-values of <0.05 were considered as statistically significant.

## Results

### Selection of Collimation Angle

**Table 5** shows the dosimetric indexes of 10 left-sided postmastectomy VMAT plans with different collimation angles. Through the comprehensive judgment of the dosimetric indexes of the plans, the selected collimation angle is 350 degrees. The average heart D_mean_ of the 350-degree collimation plans (7.41 Gy, p = 0.031) was significantly lower than that of the 5-degree collimation plans (7.59 Gy) and the average ipsilateral lung D_mean_ (13.17 Gy, p = 0.046) significantly lower than that of the 5 degree collimation plans (13.49 Gy). The average PTV HI of the 350-degree collimation plans (0.148, p = 0.030) was significantly lower than that of the 10-degree collimation plans (0.156), the average ipsilateral lung D_mean_ (13.17 Gy, p = 0.002) significantly lower than that of the 10-degree collimation plans (13.52 Gy), the average ipsilateral lung V_5Gy_ (48.7%, p < 0.001) significantly lower than that of the 10-degree collimation plans (51.6%), and the average ipsilateral lung V_20Gy_ (23.0%, p = 0.001) significantly lower than that of the 10-degree collimation plans (23.4%), and the rest of the indexes were not significantly different (p > 0.05). The above indexes of the 350-degree collimation plans are all significantly better than that of the 5- and 10-degree collimation plans, indicating that the 350-degree collimation plans are better than those of the 5- and 10-degree collimation plans. Except for spinal cord D_0.03cc_, the average dosimetric indexes of the 350-degree collimation plans is better than that of the 355-degree collimation plans, but there are no significant difference. After comprehensive consideration, the 350-degree collimation plans tend to be preferred.

**Table 5:** Dosimetric results (mean ± standard deviation) of left-sided postmastectomy VMAT plans with different collimation angles.

	350 degree	5 degree	p*^a^*	10 degree	p*^b^*	355 degree	p*^c^*
PTV CI	0.850±0.011	0.845±0.016	0.133	0.845±0.012	0.092	0.848±0.015	0.300
PTV HI	0.148±0.015	0.151±0.025	0.401	0.156±0.019	0.030	0.149±0.019	0.834
Heart D_mean_ (Gy)	7.41±1.06	7.59±1.26	0.031	7.65±1.23	0.116	7.48±1.14	0.400
Spinal cord D_0.03cc_ (Gy)	14.76±4.25	14.87±4.39	0.859	16.29±6.61	0.295	14.31±2.88	0.629
Ipsilateral lung D_mean_ (Gy)	13.17±0.72	13.49±0.97	0.046	13.52±0.85	0.002	13.27±0.86	0.356
Ipsilateral lung V_5Gy_ (%)	48.7±4.2	50.7±6.2	0.070	51.6±5.2	<0.001	49.8±5.9	0.185
Ipsilateral lung V_20Gy_ (%)	23.0±1.3	23.3±1.6	0.144	23.4±1.4	0.001	23.1±1.4	0.487
Contralateral lung D_mean_ (Gy)	2.69±0.58	2.59±0.54	0.052	2.61±0.62	0.207	2.75±0.61	0.429
Contralateral lung V_5Gy_ (%)	13.8±4.3	13.2±4.0	0.298	12.8±4.7	0.320	14.6±4.4	0.323
Lungs D_mean_ (Gy)	7.24±0.61	7.33±0.74	0.751	7.34±0.73	0.065	7.31±0.69	0.183

^a^Comparison of 350 degree to 5 degree.

^b^Comparison of 350 degree to 10 degree.

^c^Comparison of 350 degree to 355 degree.

**Table 6** shows the dosimetric indexes of 10 right-sided postmastectomy VMAT plans with different collimation angles. The selected collimation angle is 10 degrees. The average spinal cord D_0.03cc_ of the 10-degree collimation plans (15.46 Gy, p = 0.039) was significantly higher than that of the 350-degree collimation plans (12.68 Gy), the average ipsilateral lung D_mean_ (13.65 Gy, p = 0.002) significantly lower than that of the 350-degree collimation plans (13.99 Gy), the average ipsilateral lung V_5Gy_ (49.3%, p = 0.006) significantly lower than that of the 350-degree collimation plans (51.8%), and the average lungs D_mean_ (8.41 Gy, p = 0.014) significantly lower than that of the 350-degree collimation plans (8.57 Gy). The average ipsilateral lung D_mean_ of the 10-degree collimation plans (13.65 Gy, p = 0.005) was significantly lower than that of the 355-degree collimation plans (13.86 Gy), the average ipsilateral lung V_5Gy_ (49.3%, p = 0.013) significantly lower than that of the 355-degree collimation plans (51.1%), the average ipsilateral lung V_20Gy_ (24.2%, p = 0.012) significantly lower than that of the 355-degree collimation plans (24.3%), the average lungs D_mean_ (8.41 Gy, p = 0.040) significantly lower than that of the 355-degree collimator plans (8.52 Gy), and the rest of the indexes were not significantly different (p > 0.05). The above indexes of the 10-degree collimation plans are all significantly better than those of the 355-degree collimation plans, indicating that the 10-degree collimation plans are better than those of the 355-degree collimation plans. Although the average spinal cord D_0.03cc_ of the 350 degree collimation plans is better than that of the 10-degree collimation plans, after comprehensive consideration, the 10-degree collimator plans tend to be preferred. The average PTV CI, PTV HI, spinal cord D_0.03cc_, ipsilateral lung D_mean_, ipsilateral lung V_5Gy_, and lungs D_mean_ of the 10-degree collimator plans are all better than those of the 5-degree collimation plans, but there is no significant difference. After comprehensive consideration, the 10-degree collimation plans tend to be preferred.

**Table 6:** Dosimetric results (mean ± standard deviation) of right-sided postmastectomy VMAT plans with different collimation angles.

	10 degree	5 degree	p*^a^*	350 degree	p*^b^*	355 degree	p*^c^*
PTV CI	0.846±0.012	0.842±0.015	0.242	0.837±0.017	0.062	0.841±0.016	0.252
PTV HI	0.150±0.019	0.154±0.022	0.080	0.161±0.026	0.063	0.156±0.022	0.154
Heart D_mean_ (Gy)	3.47±0.81	3.36±0.81	0.101	3.39±0.90	0.507	3.46±0.91	0.094
Spinal cord D_0.03cc_ (Gy)	15.46±3.69	15.73±2.22	0.796	12.68±3.61	0.039	14.56±2.55	0.128
Ipsilateral lung D_mean_ (Gy)	13.65±0.72	13.72±0.71	0.154	13.99±0.78	0.002	13.86±0.73	0.005
Ipsilateral lung V_5Gy_ (%)	49.3±3.7	49.9±3.4	0.132	51.8±4.1	0.006	51.1±4.0	0.013
Ipsilateral lung V_20Gy_ (%)	24.2±0.9	24.2±0.9	0.624	24.3±1.0	0.093	24.3±0.8	0.012
Contralateral lung D_mean_ (Gy)	1.97±0.29	1.89±0.38	0.354	1.90±0.38	0.277	1.92±0.37	0.428
Contralateral lung V_5Gy_ (%)	7.6±2.7	7.2±3.9	0.640	6.3±2.7	0.140	6.7±3.1	0.261
Lungs D_mean_ (Gy)	8.41±0.63	8.43±0.62	0.544	8.57±0.69	0.014	8.52±0.69	0.040

^a^Comparison of 10 degree to 5 degree.

^b^Comparison of 10 degree to 350 degree.

^c^Comparison of 10 degree to 355 degree.

### Selection of Mode of Jaw Collimator

**Table 7** shows the dosimetric indexes of 10 left- and 10 right-sided postmastectomy VMAT plans with jaw collimator modes of jaw tracking and fixed jaw. Through the comprehensive judgment of the dosimetric indexes of the plans, the jaw tracking was selected. The average PTV HI of the left-sided PMRT jaw tracking plans (0.148, p = 0.006) was significantly higher than that of the fixed jaw plans (0.145), the average heart D_mean_ (7.41 Gy, p < 0.001) significantly lower than that of the fixed jaw plans (7.90 Gy), the average spinal cord D_0.03cc_ (14.76 Gy, p = 0.017) significantly lower than that of the fixed jaw plans (16.67 Gy), the average ipsilateral lung D_mean_ (13.17 Gy, p < 0.001) significantly lower than that of the fixed jaw plans (13.61 Gy), the average ipsilateral lung V_5Gy_ (48.7%, p < 0.001) significantly lower than that of the fixed jaw plans (53.2%), the average contralateral lung D_mean_ (2.69 Gy, p = 0.004) significantly lower than that of the fixed jaw plans (2.93 Gy), and the average lungs D_mean_ (7.24 Gy, p < 0.001) significantly lower than that of the fixed jaw plans (7.56 Gy), and the rest of the indexes were not significantly different (p > 0.05). Although the average PTV HI of the fixed jaw plans is better than that of the jaw tracking plans, after comprehensive consideration, the jaw tracking plans tend to be preferred.

**Table 7:** Dosimetric results (mean ± standard deviation) of postmastectomy VMAT plans with jaw tracking and fixed jaw.

	Jaw tracking (left-side)	Fixed jaw (left-side)	p (left-side)	Jaw tracking (right-side)	Fixed jaw (right-side)	p (right-side)
PTV CI	0.850±0.011	0.849±0.013	0.208	0.846±0.012	0.848±0.015	0.235
PTV HI	0.148±0.015	0.145±0.016	0.006	0.150±0.019	0.144±0.020	0.054
Heart D_mean_ (Gy)	7.41±1.06	7.90±1.10	<0.001	3.47±0.81	3.92±0.74	<0.001
Spinal cord D_0.03cc_ (Gy)	14.76±4.25	16.67±4.05	0.017	15.46±3.69	15.09±3.06	0.669
Ipsilateral lung D_mean_ (Gy)	13.17±0.72	13.61±0.73	<0.001	13.65±0.72	14.19±0.74	<0.001
Ipsilateral lung V_5Gy_ (%)	48.7±4.2	53.2±5.1	<0.001	49.3±3.7	54.2±4.5	<0.001
Ipsilateral lung V_20Gy_ (%)	23.0±1.3	23.1±1.3	0.115	24.2±0.9	24.2±1.0	0.543
Contralateral lung D_mean_ (Gy)	2.69±0.58	2.93±0.57	0.004	1.97±0.29	2.19±0.35	0.005
Contralateral lung V_5Gy_ (%)	13.8±4.3	15.2±4.3	0.175	7.6±2.7	8.5±2.8	0.041
Lungs D_mean_ (Gy)	7.24±0.61	7.56±0.64	<0.001	8.41±0.63	8.82±0.64	<0.001

The average heart D_mean_ of the right-sided PMRT jaw tracking plans (3.47 Gy, p < 0.001) was significantly lower than that of the fixed jaw plans (3.92 Gy), the average ipsilateral lung D_mean_ (13.65 Gy, p < 0.001) significantly lower than that of the fixed jaw plans (14.19 Gy), the average ipsilateral lung V_5Gy_ (49.3%, p < 0.001) significantly lower than fixed jaw plans (54.2%), the average contralateral lung D_mean_ (1.97 Gy, p = 0.005) significantly lower than fixed jaw plans (2.19 Gy), the average contralateral lung V_5Gy_ (7.6%, p = 0.041) significantly lower than fixed jaw plans (8.5%), and the average lungs D_mean_ (8.41 Gy, p < 0.001) significantly lower than fixed jaw plans (8.82 Gy), and rest of the indexes were not significantly different (p > 0.05). The above indexes of the jaw tracking plans are all significantly better than those of the fixed jaw plans, indicating that the jaw tracking plans are better than the fixed jaw plans.

### Selection of the GSR

**Table 8** shows the dosimetric indexes of the 10 left- and 10 right-sided postmastectomy VMAT plans with 2- and 4-degree GSR. Through the comprehensive judgment of the dosimetric indexes of the plans, the 2-degree GSR was selected. The average heart D_mean_ of the left-sided PMRT 2-degree GSR plans (7.41 Gy, p < 0.001) was significantly lower than that of the 4-degree GSR plans (7.66 Gy), the average ipsilateral lung D_mean_ (13.17 Gy, p = 0.002) significantly lower than that of the 4 degree GSR plans (13.34 Gy), the average ipsilateral lung V_5Gy_ (48.7%, p = 0.010) significantly lower than that of the 4 degree GSR plans (50.0%), the average ipsilateral lung V_20Gy_ (23.0%, p < 0.010) significantly lower than that of the 4-degree GSR plans (23.3%), and the average lungs D_mean_ (7.24 Gy, p = 0.014) significantly lower than that of the 4-degree GSR plans (7.32 Gy), and the rest of the indexes were not significantly different (p > 0.05). The above indexes of the 2-degree GSR plans are all significantly better than those of the 4-degree GSR plans, indicating that the 2-degree GSR plans are better than that of the 4-degree GSR plans.

**Table 8:** Dosimetric results (mean ± standard deviation) of postmastectomy VMAT plans with 2 degree and 4 degree gantry spacing resolution.

	2 degree (left-side)	4 degree (left-side)	p (left-side)	2 degree (right-side)	4 degree (right-side)	p (right-side)
PTV CI	0.850±0.011	0.849±0.010	0.089	0.846±0.012	0.841±0.014	0.013
PTV HI	0.148±0.015	0.150±0.012	0.220	0.150±0.019	0.156±0.018	0.012
Heart D_mean_ (Gy)	7.41±1.06	7.66±1.09	<0.001	3.47±0.81	3.66±0.94	0.006
Spinal cord D_0.03cc_ (Gy)	14.76±4.25	15.19±3.65	0.243	15.46±3.69	16.35±4.09	0.093
Ipsilateral lung D_mean_ (Gy)	13.17±0.72	13.34±0.69	0.002	13.65±0.72	13.78±0.64	0.002
Ipsilateral lung V_5Gy_ (%)	48.7±4.2	50.0±4.9	0.010	49.3±3.7	49.7±3.5	0.110
Ipsilateral lung V_20Gy_ (%)	23.0±1.3	23.3±1.1	<0.001	24.2±0.9	24.5±0.7	0.001
Contralateral lung D_mean_ (Gy)	2.69±0.58	2.71±0.60	0.704	1.97±0.29	2.00±0.28	0.302
Contralateral lung V_5Gy_ (%)	13.8±4.3	14.0±4.5	0.504	7.6±2.7	7.9±2.4	0.386
Lungs D_mean_ (Gy)	7.24±0.61	7.32±0.58	0.014	8.41±0.63	8.50±0.61	<0.001

The average PTV CI of the right-sided PMRT 2-degree GSR plans (0.846, p = 0.013) was significantly higher than that of the 4-degree GSR plans (0.841), and the average PTV HI (0.150, p = 0.012) is significantly lower than that of the 4-degree GSR plans (0.156), the average heart D_mean_ (3.47 Gy, p = 0.006) significantly lower than that of the 4-degree GSR plans (3.66 Gy), the average ipsilateral lung D_mean_ (13.65 Gy, p = 0.002) significantly lower than that of the 4-degree GSR plan (13.78 Gy), the average ipsilateral lung V_20Gy_ (24.2%, p = 0.001) significantly lower than that of the 4-degree GSR plan (24.5%), and the average lungs D_mean_ (8.41 Gy, p < 0.001) significantly lower than that of the 4-degree GSR plans (8.50 Gy), and the rest of the indexes were not significantly different (p > 0.05). The above indexes of the 2-degree GSR plans are all significantly better than those of the 4-degree GSR plans, indicating that the 2-degree GSR plans are better than that of the 4-degree GSR plans.

### Selection of the Number of Start Optimization Times

**Table 9** shows the dosimetric indexes of the 10 left- and 10 right-sided postmastectomy VMAT plans with 2 rounds of optimization and 3 rounds of optimization. **Table 10** shows the iterations completed in each round of optimization for the 10 left- and 10 right-sided PMRT cases. The maximum 100 iterations per round were completed in the first 2 rounds of optimization for almost all cases, while the third round of optimization were not completed for most cases as the iteration stopping tolerance was reached. Through the comprehensive judgment of the dosimetric indexes of the plans, the 3 rounds of optimization were selected. The average PTV CI of the left-sided PMRT plans with 2 rounds of optimization (0.850, p = 0.002) was significantly lower than that of the plans with 3 rounds of optimization (0.854), and the average PTV HI (0.148, p = 0.003) was significantly higher than that of the plans with 3 rounds of optimization (0.143), the average heart D_mean_ (7.41 Gy, p = 0.003) significantly higher than that of the plans with 3 rounds of optimization (7.16 Gy), the average spinal cord D_0.03cc_ (14.76 Gy, p < 0.001) significantly higher than that of the plans with 3 rounds of optimization (14.14 Gy), the average ipsilateral lung D_mean_ (13.17 Gy, p = 0.001) significantly higher than that of the plans with 3 rounds of optimization (12.95 Gy), the average ipsilateral lung V_5Gy_ (48.7%, p = 0.009) significantly higher than that of the plans with 3 rounds of optimization (47.4%), the average ipsilateral lung V_20Gy_ (23.0%, p < 0.001) significantly higher than that of the plans with 3 rounds of optimization (22.5%), the average contralateral lung V_5Gy_ (13.8%, p = 0.020) significantly lower than that of the plans with 3 rounds of optimization (14.3%), and the average lungs D_mean_ (7.24 Gy, p < 0.001) significantly higher than that of the plans with 3 rounds of optimization (7.17 Gy), and the rest of the indexes were not significantly different (p > 0.05). Although the average contralateral lung V_5Gy_ of the plans with 2 rounds of optimization is better than that of the plans with 3 rounds of optimization, after comprehensive consideration, the plans with 3 rounds of optimization tend to be preferred.

**Table 9:** Dosimetric results (mean ± standard deviation) of postmastectomy VMAT plans with 2 round and 3 round optimizations.

	2 round (left-side)	3 round (left-side)	p (left-side)	2 round (right-side)	3 round (right-side)	p (right-side)
PTV CI	0.850±0.011	0.854±0.012	0.002	0.846±0.012	0.851±0.011	0.027
PTV HI	0.148±0.015	0.143±0.014	0.003	0.150±0.019	0.142±0.015	0.002
Heart D_mean_ (Gy)	7.41±1.06	7.16±0.93	0.003	3.47±0.81	3.41±0.74	0.055
Spinal cord D_0.03cc_ (Gy)	14.76±4.25	14.14±4.21	<0.001	15.46±3.69	14.89±4.03	0.025
Ipsilateral lung D_mean_ (Gy)	13.17±0.72	12.95±0.72	0.001	13.65±0.72	13.53±0.63	0.010
Ipsilateral lung V_5Gy_ (%)	48.7±4.2	47.4±3.6	0.009	49.3±3.7	48.4±3.3	0.006
Ipsilateral lung V_20Gy_ (%)	23.0±1.3	22.5±1.4	<0.001	24.2±0.9	23.9±0.8	0.001
Contralateral lung D_mean_ (Gy)	2.69±0.58	2.74±0.60	0.065	1.97±0.29	1.95±0.32	0.626
Contralateral lung V_5Gy_ (%)	13.8±4.3	14.3±4.3	0.020	7.6±2.7	7.7±2.5	0.591
Lungs D_mean_ (Gy)	7.24±0.61	7.17±0.62	<0.001	8.41±0.63	8.34±0.59	0.025

**Table 10:** Iterations completed in each round of optimization of postmastectomy VMAT plans.

Case no.	1st round (left-side)	2nd round (left-side)	3rd round (left-side)	1st round (right-side)	2nd round (right-side)	3rd round (right-side)
1	100	100	10	100	100	100
2	100	93	31	100	100	38
3	100	100	93	100	100	93
4	100	100	65	100	100	93
5	100	100	100	100	100	12
6	100	100	93	100	100	12
7	100	100	26	100	100	100
8	100	100	100	100	100	12
9	100	100	100	100	100	93
10	100	100	100	100	100	93

The average PTV CI of the right-sided PMRT plans with 2 rounds of optimization (0.846, p = 0.027) was significantly lower than that of the plans with 3 rounds of optimization (0.851), the average PTV HI (0.150, p = 0.002) significantly higher than that of the plans with 3 rounds of optimization (0.142), the average spinal cord D_0.03cc_ (15.46 Gy, p = 0.025) significantly higher than that of the plans with 3 rounds of optimization (14.89 Gy), the average ipsilateral lung D_mean_ (13.65 Gy, p = 0.010) significantly higher than that of the plans with 3 rounds of optimization (13.53 Gy), the average ipsilateral lung V_5Gy_ (49.3%, p = 0.006) significantly higher than that of the plans with 3 rounds of optimization (48.4%), the average ipsilateral lung V_20Gy_ (24.2%, p = 0.001) significantly higher than that of the plans with 3 rounds of optimization (23.9%), and the average lungs D_mean_ (8.41 Gy, p = 0.025) significantly higher than that of the plans with 3 rounds of optimization (8.34 Gy), and the rest of the indexes were not significantly different (p > 0.05). The above indexes of plans with 3 rounds of optimization are all significantly better than that of the plans with 2 rounds of optimization, indicating that the plans with 3 rounds of optimization are better than those of the plans with 2 rounds of optimization.

### Automated Treatment Planning

**Figure 1:**
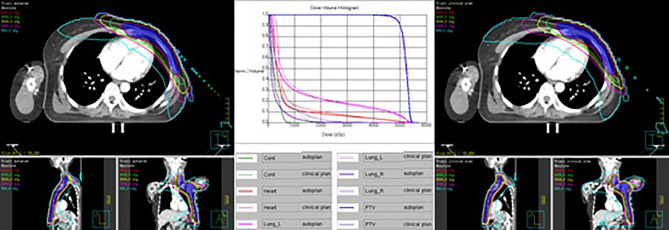
Dose distribution and DVH for autoplan (left) and clinical plan (right) of 1 left-sided PMRT case. The blue colorwash area is PTV. The medium solid lines are isodose lines.

**Figure 2:**
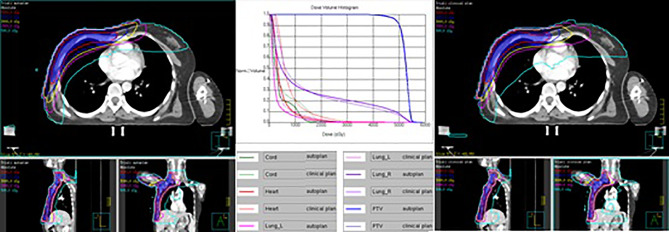
Dose distribution and DVH for autoplan (left) and clinical plan (right) of 1 right-sided PMRT case. The blue colorwash area is PTV. The medium solid lines are isodose lines.

**Table 11** shows the dosimetric index of 10 left- and 10 right-sided postmastectomy VMAT autoplans and manual clinical plans. **Figure 1** shows the dose distribution and DVH for the autoplan and clinical plan of 1 left-sided PMRT case. The left picture in **Figure 1** is the autoplan, and the right one is the clinical plan. The blue colorwash area is PTV. The medium solid lines are isodose lines. Similarly, **Figure 2** shows the dose distribution and DVH for the autoplan and clinical plan of 1 right-sided PMRT case. The average PTV CI of the left-sided PMRT autoplans (0.854, p = 0.042) was significantly higher than that of clinical plans (0.812), the average heart D_mean_ (7.16 Gy, p < 0.001) significantly lower than that of clinical plans (9.75 Gy), the average spinal cord D_0.03cc_ (14.14 Gy, p = 0.012) significantly lower than that of the clinical plans (22.89 Gy), the average ipsilateral lung D_mean_ (12.95 Gy, p = 0.012) significantly lower than clinical plans (14.02 Gy), the average ipsilateral lung V_5Gy_ (47.4%, p < 0.001) significantly lower than that of the clinical plans (58.2%), the average contralateral lung D_mean_ (2.74 Gy, p = 0.012) significantly lower than that of the clinical plans (4.32 Gy), the average contralateral lung V_5Gy_ (14.3%, p = 0.032) significantly lower than that of the clinical plans (23.3%), and the average lungs D_mean_ (7.17 Gy, p < 0.001) significantly lower than that of the clinical plans (8.53 Gy), and the rest of the indexes were not significantly different (p > 0.05). All of the above indexes of autoplans are significantly better than those of the manual clinical plans, indicating that autoplans are better than manual clinical plans.

**Table 11:** Dosimetric results (mean ± standard deviation) of postmastectomy VMAT autoplans and clinical plans.

	Autoplan (left-side)	Clinical plan (left-side)	P (left-side)	Autoplan (right-side)	Clinical plan (right-side)	P (right-side)
PTV CI	0.854±0.012	0.812±0.056	0.042	0.851±0.011	0.842±0.009	0.002
PTV HI	0.143±0.014	0.204±0.103	0.083	0.142±0.015	0.169±0.027	0.004
Heart D_mean_ (Gy)	7.16±0.93	9.75±1.17	<0.001	3.41±0.74	7.88±1.70	<0.001
Spinal cord D_0.03cc_ (Gy)	14.14±4.21	22.89±9.61	0.012	14.89±4.03	21.04±5.65	0.019
Ipsilateral lung D_mean_ (Gy)	12.95±0.72	14.02±0.92	0.012	13.53±0.63	14.17±0.46	0.003
Ipsilateral lung V_5Gy_ (%)	47.4±3.6	58.2±5.9	<0.001	48.4±3.3	57.9±4.2	<0.001
Ipsilateral lung V_20Gy_ (%)	22.5±1.4	23.8±2.4	0.197	23.9±0.8	24.2±1.2	0.644
Contralateral lung D_mean_ (Gy)	2.74±0.60	4.32±1.73	0.012	1.95±0.32	4.03±0.63	<0.001
Contralateral lung V_5Gy_ (%)	14.3±4.3	23.3±11.8	0.032	7.7±2.5	20.2±7.4	<0.001
Lungs D_mean_ (Gy)	7.17±0.62	8.53±0.91	<0.001	8.34±0.59	9.62±0.58	<0.001

The average PTV CI of the right-sided PMRT autoplans (0.851, p = 0.002) was significantly higher than that of the clinical plans (0.842), the average PTV HI (0.142, p = 0.004) significantly lower than that of the clinical plans (0.169), the average heart D_mean_ (3.41 Gy) significantly lower than that of the clinical plans (7.88 Gy, p < 0.001), the average spinal cord D_0.03cc_ (14.89 Gy, p=0.019) significantly lower than that of the clinical plans (21.04 Gy), the average ipsilateral lung D_mean_ (13.53 Gy, p = 0.003) significantly lower than that of the clinical plans (14.17 Gy), the average ipsilateral lung V_5Gy_ (48.4%, p < 0.001) significantly lower than that of the clinical plans (57.9%), the average contralateral lung D_mean_ (1.95 Gy, p < 0.001) significantly lower than that of the clinical plans (4.03 Gy), the average contralateral lung V_5Gy_ (7.7%, p < 0.001) significantly lower than that of the clinical plans (20.2%), and the average lungs D_mean_ (8.34 Gy, p < 0.001) significantly lower than that of the clinical plans (9.62 Gy), and the rest of the indexes were not significantly different (p > 0.05). All of the above indexes of autoplans are significantly better than those of the manual clinical plans, indicating that autoplans are better than manual clinical plans.

## Discussions

In this study, the MDAP system and the Pinnacle TPS were used to implement and evaluate an artificial intelligence-based automated treatment planning method for postmastectomy VMAT, which achieved better plan quality than manual clinical plans. To the best of our knowledge, this is the first time that the automated treatment planning for left- and right-sided postmastectomy VMAT is fully realized, and this is the first time that the treatment planning parameters such as collimation angle, jaw collimator mode, GSR, and number of start optimization times were investigated in the automated treatment planning study.

Similar to the study by Zhang et al. (13), the setting of optimization objectives in this study included the equivalent uniform dose (EUD) constraint, which reduces the dose to organs at risk and normal tissues more effectively. The constraint of maximum EUD in the optimization objective template of this study is set to 0, and only the weight and the value a need to be set, which is more conducive to ensure the generalization of the automated treatment planning.

Referring to the setting of the gantry angle range of Zhang et al. (13) and Cilla et al. (17), since the cases in this study included the internal mammary region, and higher generalization is required for automated treatment planning, the setting of the gantry angle range is slightly larger, ranging from 294 to 180 degrees for the left-sided postmastectomy VMAT plans and 181 to 66 degrees for the right-sided postmastectomy VMAT plans. Because the dose rate and field aperture at each gantry angle can be well modulated for VMAT, and the jaw tracking mode is used to reduce the leakage dose, the low-dose volume can be controlled well while ensuring the dose conformity and uniformity of the target volume.

The size of the jaw collimator at each gantry angle and the field aperture formed by the MLC are affected by the collimation angle. Increasing the width of the jaw collimator in the x-direction may increase the leakage dose and affect the degree of freedom of the movement of MLC. When the collimation angle deviates more from 0 degrees, the width of the jaw collimator in the x-direction at some gantry angles may increase. Therefore, the collimation angle range selected in this study is plus or minus 5 degree and 10 degrees from 0 degree. Due to the different spatial relationship between the left- and right-sided PMRT target volume and the organ at risk, the good collimation angle may also be different. In this study, the 350-degree collimation angle was selected for the left-sided postmastectomy VMAT plan, and the 10-degree collimation angle was selected for the right-sided postmastectomy VMAT plan.

Theoretically, jaw tracking is beneficial to reduce the leakage dose, which has been confirmed in several studies (23, 24). In this study, the jaw tracking technique was chosen for the left- and right-sided postmastectomy VMAT plans. The maximum distance of the Millennium 120 MLC of the Varian accelerator which extends out of the carriage is 14–15 cm. In order to avoid the excessive size of the jaw collimator and affect the optimization of the field aperture formed by the MLC, the maximum size limit of the x-direction movement of the jaw collimator was set to be 10 cm to the left and right in this study.

The Pinnacle TPS provides a choice of 2–4 degrees for GSR. In theory, the 2-degree GSR provides more degrees of freedom for the optimization of treatment planning than 4 degrees. The 4-degree GSR plan is a simple special case of the 2-degree GSR plan; that is, a 4-degree GSR plan is equivalent to the 2-degree GSR plan generated by linear interpolating the beam parameters of two adjacent control points in the middle of each 4-degree interval. This study first demonstrated the dosimetric advantage of 2-degree GSR over 4-degree GSR for left- and right-sided postmastectomy VMAT plans.

In theory, more optimization iterations are beneficial to get a plan closer to the optimization objectives, but it will also take up more computing and time resources. In this study, for most of the cases, the very strict iteration stopping tolerance was reached without completing the 100 iterations in the third round of optimization, so this study did not involve more comparison and selection of the number of start optimization times, and too many optimization iterations may increase excessive plan complexity. In this study, we chose to start optimization 3 times for the left- and right-sided postmastectomy VMAT plans.

In this study, the MDAP system was used to realize automated treatment planning, which has been routinely used in clinical practice in our hospital. From the data in **Table 11**, it can be seen that most of the dosimetric indexes of the autoplans are significantly better than those of the manual clinical plans. Compared with manual clinical plans, autoplans significantly improved PTV CI, reduced the mean heart dose, mean lung dose, and lung V_5Gy_, and right-sided postmastectomy VMAT autoplans significantly reduced PTV HI, thus reducing the toxicity and side effects of normal tissues, skin reactions, the probability of radiation pneumonitis, and especially the probability of coronary events in the heart (25).

Using the MDAP system to generate an autoplan takes about 1 h, and almost no manual intervention is required, so the computing and human resources are not occupied much, which can improve clinical efficiency and it is suitable for clinical treatment.

There are still some limitations in this study. First, the study did not include cases using the deep-inspiration breath-hold technique (26), and all cases included the internal mammary region. Although in theory, the use of the deep-inspiration breath-hold technique or without the internal mammary region is beneficial to the protection of organs at risk such as the heart, which can reduce the difficulty of treatment planning, and the automated treatment planning method is also applicable, it still needs further research to confirm or make modifications. Secondly, the cases selected in this study did not delineate the organs at risk such as the contralateral breast, larynx, trachea, esophagus, thyroid, liver, stomach, and intestines. With the popularization and application of the automated delineation system, we will complete the delineation and increase the optimization objectives in the next study, which may have a small impact on the optimization results of the autoplan, and the automated treatment planning method needs to be confirmed or modified.

## Conclusions

In this study, the MDAP system and the Pinnacle TPS were used to implement and evaluate an artificial intelligence-based automated planning method for postmastectomy VMAT, which achieved better plan quality than the manual clinical plan, and improved clinical efficiency.

## Data Availability Statement

The original contributions presented in the study are included in the article/supplementary material. Further inquiries can be directed to the corresponding author.

## Author Contributions

Design of the study—SJ, XZ, WW. Acquisition of data—SJ, ML, CY, JW, JC, JY. Analysis and interpretation of data—SJ, YX. Drafting and revising of the article—SJ, YX, ML, CY, DZ, QW, ZY, XW, XZ, WW. Review and approval of the manuscript—all. All authors contributed to the article and approved the submitted version.

## Funding

Using the mdaccAutoPlan system to improve radiotherapy plan quality while reducing cost', Sister Institution Network Fund (SINF), Global Academic Programs, MD Anderson Cancer Center.

## Conflict of Interest

The authors declare that the research was conducted in the absence of any commercial or financial relationships that could be construed as a potential conflict of interest.

## Publisher’s Note

All claims expressed in this article are solely those of the authors and do not necessarily represent those of their affiliated organizations, or those of the publisher, the editors and the reviewers. Any product that may be evaluated in this article, or claim that may be made by its manufacturer, is not guaranteed or endorsed by the publisher.
